# Assessing the efficiency of Iran health system in making progress towards universal health coverage: a comparative panel data analysis

**DOI:** 10.1186/s12962-020-00215-x

**Published:** 2020-06-29

**Authors:** Haniye Sadat Sajadi, Zahra Goodarzi, Amirhossein Takian, Efat Mohamadi, Alireza Olyaeemanesh, Farhad Hosseinzadeh Lotfi, Hamid Sharafi, Somayeh Noori Hekmat, Matthew Jowett, Reza Majdzadeh

**Affiliations:** 1 Knowledge Utilization Research Center, University Research and Development Center, Tehran University of Medical Sciences, Tehran, Iran; 2 National Institute for Health Research, Tehran University of Medical Sciences, Tehran, Iran; 3 Department of Global Health & Public Policy, Department of Management Sciences & Health Economics, School of Public Health, Health Equity Research Center, Tehran University of Medical Sciences, Tehran, Iran; 4 Health Equity Research Center, Tehran University of Medical Sciences, Tehran, Iran; 5 National Institute for Health Research, Health Equity Research Center, Tehran University of Medical Sciences, Tehran, Iran; 6 Department of Mathematics, Science and Research Branch, Islamic Azad University, Tehran, Iran; 7 Management and Leadership in Medical Education Research Center, Kerman University of Medical Sciences, Kerman, Iran; 8 Department of Health Systems Governance & Financing, World Health Organization, Geneva, Switzerland; 9 Knowledge Utilization Research Center, Community-Based Participatory-Research Center,and School of Public Health, Tehran University of Medical Sciences, Tehran, Iran

**Keywords:** Efficiency, Productivity, Health system, Health reform, Universal health coverage, Iran

## Abstract

**Background:**

Building upon decades of continuous reforms, Iran has been implementing various initiatives to reach universal health coverage (UHC). Improving efficiency is a crucial intermediate policy objective for UHC. Therefore, this article aimed to measure the efficiency and productivity changes of the Iranian health system in making progress towards UHC during 2010−2015 in comparison with 36 selected other upper-middle-income countries.

**Methods:**

We used panel data to measure the variations in technical efficiency (TE) and total factor productivity (TFP) through an extended data envelopment analysis (EDEA) and Malmquist productivity index, respectively. General government health expenditure (GGHE) per capita (International dollar) was selected as the input variable. Service coverage of diphtheria, tetanus and pertussis; family planning; antiretroviral therapy; skilled attendants at birth; Tuberculosis treatment success rate; and GGHE as  % of total health expenditure (THE) were considered as output variables. The data for each indicator were taken from the Global Health Observatory data repository and World Development Indicator database, for 6 years (2010−2015).

**Results:**

The TE scores of Iran’s health system were 0.75, 0.77, 0.74, 0.74, 0.97, and 0.84 in the period 2010–2015, respectively. TFP improved in 2011 (1.02), 2013 (1.01), and 2014 (1.30, generally). The overall efficiency and TFP increased in 2014. Changes made in CCHE per capita and GGHE/THE attributed to the increase of efficiency.

**Conclusion:**

There is a growing demand for efficiency improvements in the health systems to achieve UHC. While there are no defined set of indicators or precise methods to measure health system efficiency, EDEA helped us to draw the picture of health system efficiency in Iran. Our findings highlighted the essential need for targeted and sustained interventions, i.e., allocation of enough proportion of public funds to the health sector, to improve universal financial coverage against health costs aiming to enhance the future performance of Iran’s health system, ultimately. Such tailored interventions may also be useful for settings with similar context to speed up their movement towards improving efficiency, which in turn might lead to more resources to reach UHC.

## Background

Highly emphasized by World Health Organization (WHO), Universal Health Coverage (UHC) has become a global priority for many countries over the past decade [1, 2], rendering major health sector reforms [3–7] that might require additional financial resources in their settings [8, 9]. Given financial constraints in most countries due to the growing health care needs, particularly in the Low and Middle-Income Countries (LMICs), the fiscal space needed to achieve UHC mainly depends on savings derived from efficiency measures, which in turn relates to improvements in the health system performance [9–11]. WHO advocates greater spending more efficiently and equitably to enhance health coverage, increase financial protection and improve health outcomes [2, 12, 13]. It is estimated that between 20 and 40% of health spending is wasted, whereas many people are severely deprived of needed care, globally [2, 14]. Hence, waste reduction in the health systems has been a pivotal concern for health policymakers and managers. As a result, ensuring efficiency improvement in the health systems is crucial to optimize the use of limited health inputs.

Health system efficiency is the extent to which the inputs to the health system are used to secure valued health system outputs. Two main concepts exist for efficiency: Allocative Efficiency (AE) and Technical Efficiency (TE). AE refers to scrutinize either the choice of outputs or the choice of inputs. It determines whether limited resources are directed towards producing the correct mix of health outputs. AE also examines whether the entity uses an optimal mix of inputs to produce its chosen outputs, given the prices of those inputs [10]. TE indicates the extent to which the health system is maximizing outputs for a given set of inputs or minimizing inputs for a given set of outputs [15]. In this study, we use the term efficiency as TE. Major policy changes, epidemiological transitions, changes in the government, climate change, etc., might have a positive or negative impact on health system performance [16]. Thus, to measure variations in health system performance, we measured productivity growth over time, which might be an opportunity to improve public welfare [17].

As in the course of last four decades, The Islamic Republic of Iran has been initiating a number of health systems reforms, i.e., the establishment of an extensive Primary Health Care (PHC) network, the family physician program, and recently Health Transformation Plan (HTP), to pave the way towards UHC [18, 19]. These reforms resulted in significant achievements in strengthening the health system. For example, following HTP, the share of health expenditures from the gross domestic product increased from 6.5% in 2012 to 8.9% in 2015. The share of public expenditures from THE also increased from 33.3% in 2012 to 51.3 in 2015. Nevertheless, several challenges continued to exist, the most important of which is inefficiency in the health system [20]. Health costs will be raised; the economic status is not promising [21, 22], which makes the implementation of “resilient economy” policies inevitable [23], now more than ever [24, 25]. Hence, it is essential to measure the health system performance variations within two main dimensions of UHC (service coverage and financial protection), in relation to levels of health spending, and to make the health system more efficient as to ultimately convinced the government and the public that the highest cost is worth the value.

To the best of our knowledge, previous studies on the extent of efficiency of health systems have mainly focused on health outcomes [26–28]. Limited studies have been conducted to compare the performance of national health systems in making progress towards UHC goals [8, 29]. Furthermore, despite a considerable body of literature to measure healthcare efficiency at the intuitional and system levels [30–37], limited evidence exists on efficiency and productivity trends over time at the system level [8]. Therefore, this study aims: 1) to measure the TE changes and 2) to analyze productivity changes in Iran’s health system to achieve UHC goals over 2010−2015. Our findings can, we envisage, provide evidence to identify the areas in need of greater concern towards gearing up current attempts to reach UHC in Iran, and perhaps beyond. They are also used to compare the performance of health systems with similar levels of public spending in producing UHC goals. It will be of interest to the minister of health, health policymakers, parliament representatives, health financing practitioners, and researchers involved in health sector reforms.

## Methods

To overcome some limits of conventional DEA in measuring efficiency, our team created an extended DEA (EDEA). We adopted a method to analyze UHC performance relative to health spending [8] and applied panel data approach. The sample (or Decision-Making Units: DMUs) were selected 56 countries, classified as Upper Middle-Income Countries (UMICs) by the World Bank (with 896–12055 $ GDP per capita in 2015). EDEA necessitated weighted DMUs; therefore, we removed 20 countries with less than 1300,000 populations, assuming that the efficient management of the health system in populated countries would be more challenging. The population of removed countries had the most variations with the population of remaining countries. Table 1 presents the 36 (out of 56) countries and their selected specifications that were included in our study.

**Table 1:** The characteristics of selected countries

	Population (Million)	Gross domestic production per capita (PPP)	Human Development Index
Albania	9.6	15,847	0.76
Algeria	43.4	18,934	0.82
Argentina	55.3	12,295	0.79
Azerbaijan	2.9	11,803	0.78
Belarus	39.3	13,914	0.75
Bosnia and Herzegovina	16.61	10,582	0.75
Botswana	206	14,103	0.76
Brazil	9.5	17,168	0.81
Bulgaria	7.2	18,563	0.81
China	2.2	15,807	0.72
Colombia	3.5	11,714	0.77
Costa Rica	6.6	8827	0.7
Cuba	4	22,267	0.79
Dominican Republic	31.4	12,237	0.75
Ecuador	68.7	16,278	0.75
Gabon	5.6	16,389	0.71
Iran	78.3	25,129	0.79
Iraq	2.9	8194	0.73
Jamaica	79.4	19,083	0.8
Kazakhstan	10.5	14,601	0.74
Lebanon	1397	15,309	0.75
Macedonia	143.1	24,766	0.82
Malaysia	19.9	23,313	0.81
Mexico	7.1	14,049	0.79
Namibia	36.1	15,664	0.69
Panama	17.7	24,056	0.8
Paraguay	4.9	15,525	0.79
Peru	48.2	13,255	0.75
Romania	11.5	–^a^	0.78
Russia	1.9	16,562	0.7
Serbia	5.19	13,368	0.76
South Africa	30.7	26,808	0.8
Thailand	2.1	13,111	0.76
Turkey	125.9	17,336	0.77
Turkmenistan	2.4	9542	0.65
Venezuela	31.2	16,745	0.76

^a^No data was reported

A crucial step in carrying out a TE study is the selection of the most appropriate health or healthcare production input and output variables. Such a choice will be influenced by a number of factors, i.e., the availability of reliable information and the desired outputs. Since we measured the efficiency of the health system in making progress towards UHC, we chose the input and output variables from UHC tracer indicators. Given the critical role of public revenues to progress towards UHC, we considered the country’s level of public spending on health and its progress in terms of both service coverage and financial protection. Our input variable was General Government Health Expenditure (GGHE) per capita (International dollar), while we selected five (out of eight) core tracer indicators to measure progress toward UHC as output variables, i.e., service coverage of Diphtheria, Tetanus and Pertussis (DPT3); service coverage of Family Planning (FP); service coverage of Antiretroviral Therapy (ART); service coverage of Skilled Attendants at Birth (SAB) and service coverage of tuberculosis (TB) treatment success rate. We excluded the two indicators: improved water and improved sanitation, because public spending on health generally does not usually pay for these interventions, and excluded antenatal care coverage as well due to lack of official data for several countries included in this study. The last output variable in terms of financial protection was General Government Health Expenditure as a percentage of Total Health Expenditure (GGHE/THE), which is the indirect measure of financial protection. Our rationale for selecting variables was along the lines used by scholars to assess health system efficiency in making progress towards UHC [8, 29, 38]. We convened a panel of six recognized national experts to discuss the relevancy of selected variables to our analysis and managed to reach consensus on a guideline for each variable to ensure consistency in data definition and gathering. These experts were selected from public health (= 1), health economics (= 1), epidemiology (= 1), health services management (= 1), health policy (= 1), and health information system (= 1) disciplines.

We took data for each indicator from the WHO’s Global Health Observatory (GHO) data repository [39] and World Bank (WB)’s World Development Indicator database [40], for a period of 6 years (2010–2015). Since some values were missing, we applied an imputation technique to prepare data for analysis (Additional file 1). To do this, we selected a standard value for each variable to measure the difference between actual and standard values. We then imposed a penalty on any DMU if the actual value was far from a standard range. The variables were also ranked according to their importance. The standard range and weight of the variables were defined by the expert panel, as mentioned earlier (Table 2).

**Table 2:** The characteristics of variables

Variable	Standard value	Importance
Input		
General Government Health Expenditure (GGHE) per capita (International dollar)	620	Important
Outputs		
Diphtheria, Tetanus and Pertussis (DPT3) coverage	99	Important
Family Planning (FP) coverage	99	Not important
Tuberculosis (TB) success rate	99	Important
Antiretroviral Therapy (ART) coverage	< 90	Relative important
Skilled Attendants at Birth (SAB) coverage	99	Important
General Government Health Expenditure as % of Total Health Expenditure (GGHE/THE)	< 70	Very important

To examine the TE changes (Aim 1), we used extended data envelopment analysis (EDEA). EDEA determines conventionally how well a DMU, in our case a country, converts a set of inputs into a set of outputs. It assumes any deviations of DMUs from the frontier are due to technical inefficiency. A frontier is representing the efficient level of output (y) that can be produced from a given level of input (x). Therefore, DMUs are constrained to lie between completely efficient (= 1) and inefficient (= 0). Among different approaches, an output-oriented DEA model with variable returns to scale was selected.

To capture TFP changes (Aim 2), we used the Malmquist Productivity Index (MPI) that is derived from the comparison of efficiency changes (Catch-up) to Frontier-shift [41]. Since the frontiers can shift over time, when inefficiency is assumed to exist, the relative movement of any given MUI over time will, therefore, depend on both its position relative to the corresponding frontier (efficiency change) and the position of the frontier itself (technology change). The TFP >  1 means that the productivity is improved.

## Results

The TE scores of Iran’s health system were 0.75, 0.77, 0.74, 0.74, 0.97, and 0.84 in the period 2010–2015, respectively. TFP improved in 2011 (1.02), 2013 (1.01), and 2014 (1.30, generally). More details are presented below.

**Fig. 1:**
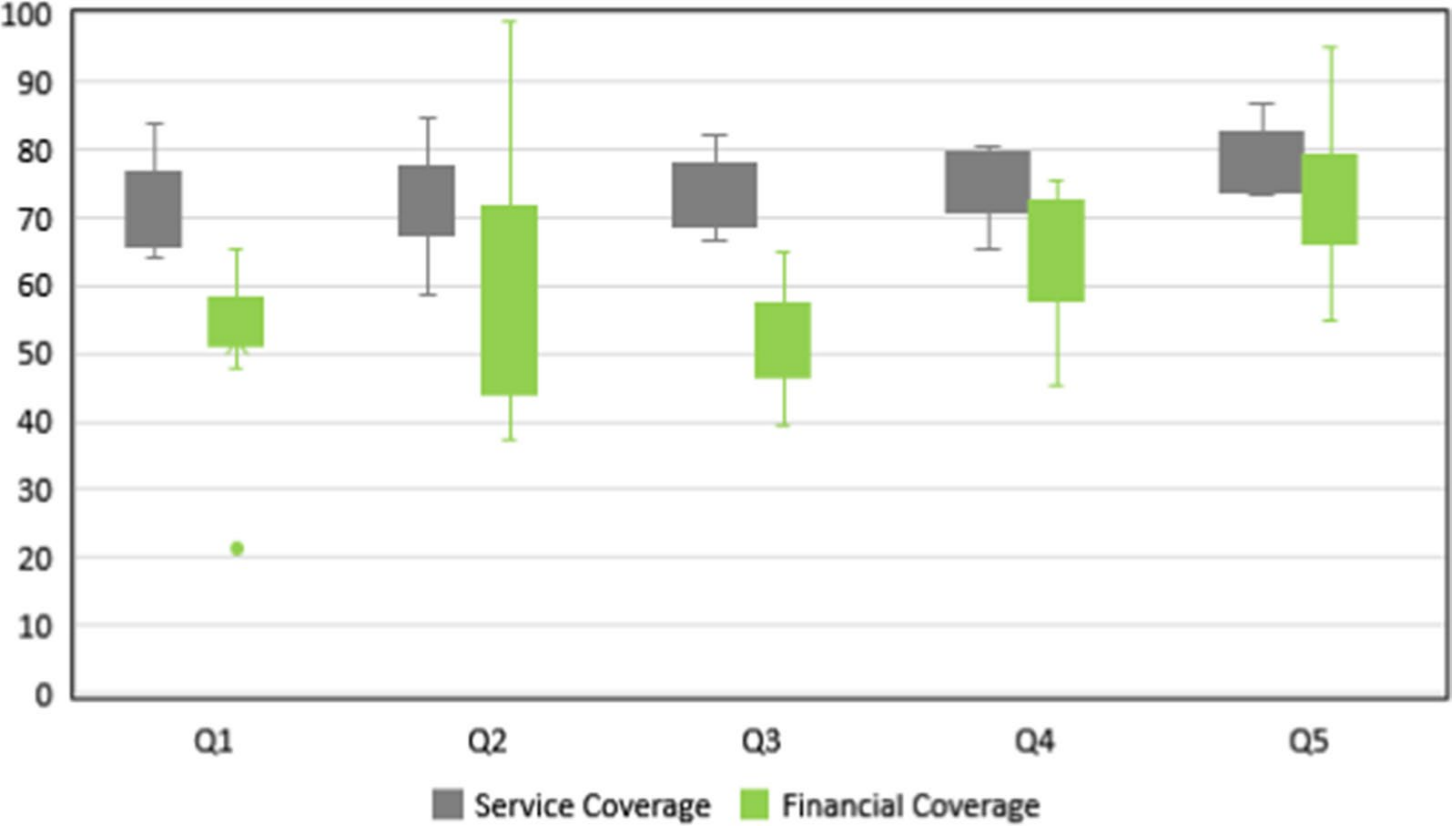
Descriptive statistics (inputs and outputs) by public spending on health quintile
B) Efficiency changes
  Table 4 reports statistics of the efficiency score of the 36 countries’ health care systems for the study period. Between 2010 and 2015, the highest efficiency score was 0.97 in 2014 in Iran.

A) Descriptive analysis
  Table 3 provides descriptive summaries of the inputs and outputs of studied countries during 2020–2015. Descriptive statistics for both service coverage and financial protection is shown in Fig. 1, in which the 36 countries are categorized into quintiles based on their level of per capita public spending on health.

**Table 3:** Descriptive summaries of the selected variables of countries during 2020–2015

Variable		Year					
		2010	2011	2012	2013	2014	2015
GGHE per capita (International dollar)	Total Iran	502 ± 298^a^ 415	519 ± 313 432	545 ± 275 401	580 ± 314 408	619 ± 377 642	593 ± 303 505
DPT3 coverage	Total Iran	91 ± 8 99	91 ± 8 99	91 ± 8 99	91 ± 8 98	90 ± 8 99	91 ± 9 98
FP coverage	Total Iran	67 ± 21 74	68 ± 21 75	68 ± 21 75	68 ± 21 75	69 ± 20 76	69 ± 20 76
TB success coverage	Total Iran	78 ± 13 83	78 ± 12 84	80 ± 9 87	80 ± 9 87	79 ± 14 87	80 ± 10 86
ART coverage	Total Iran	28 ± 15 3	31 ± 14 4	34 ± 14 6	38 ± 14 7	41 ± 14 9	47 ± 15 11
SAB coverage	Total Iran	95 ± 5 96	96 ± 4 96	97 ± 4 97	97 ± 4 97	97 ± 4 97.2	97 ± 4 96.8
GGHE as % of THE	Total Iran	60 ± 17 34	61 ± 16 35	61 ± 16 35	61 ± 16 39	61 ± 16 50	60 ± 16 43

*GGHE* General Government Health Expenditure, *DPT3* Diphtheria, Tetanus and Pertussis, *FP* Family Planning, *TB* Tuberculosis, *ART* Antiretroviral Therapy, *SAB* Skilled Attendants at Birth; THE: Total Health Expenditure

^a^Mean ± standard deviation

**Table 4:** Efficiency score of the 36 selected countries

	2010	2011	2012	2013	2014	2015	2010–2015
Albania	0.61	0.68	0.64	0.65	0.68	0.64	0.65
Algeria	0.81	0.96	1.00	1.00	1.00	1.00	0.96
Argentina	0.76	0.80	0.93	0.96	1.00	0.62	0.85
Azerbaijan	0.58	0.58	0.59	0.59	0.60	0.56	0.59
Belarus	1.00	1.00	0.99	0.88	0.99	0.91	0.96
Bosnia and Herzegovina	1.00	1.00	0.93	0.97	1.00	0.91	0.97
Botswana	0.84	0.83	0.91	0.88	0.90	1.00	0.89
Brazil	0.88	0.89	0.91	0.97	0.98	0.90	0.92
Bulgaria	0.98	0.99	0.97	0.94	0.82	0.88	0.93
China	0.62	0.71	0.68	0.71	0.80	0.60	0.69
Colombia	0.89	1.00	1.00	0.98	0.97	0.85	0.95
Costa Rica	0.73	0.76	0.66	0.64	0.68	0.63	0.68
Cuba	0.46	0.44	0.49	0.44	0.36	0.49	0.45
Dominican Republic	0.63	0.68	0.67	0.68	0.77	0.65	0.68
Ecuador	0.61	0.63	0.67	0.76	0.85	0.63	0.69
Gabon	0.73	0.70	0.73	0.85	0.77	0.77	0.76
Iran	0.75	0.77	0.74	0.74	0.97	0.84	0.80
Iraq	0.68	0.75	0.74	0.82	0.75	0.64	0.73
Jamaica	0.62	0.62	0.64	0.66	0.62	0.72	0.65
Kazakhstan	0.82	0.83	0.87	0.84	0.98	0.92	0.88
Lebanon	0.77	0.77	0.84	0.81	0.80	0.81	0.80
Macedonia	0.84	0.93	0.87	0.87	0.94	0.87	0.89
Malaysia	0.79	0.80	0.84	0.86	0.95	0.94	0.86
Mexico	0.81	0.87	0.88	0.90	0.94	0.96	0.89
Namibia	0.94	0.97	0.93	0.96	1.00	1.00	0.97
Panama	0.74	0.79	0.68	0.54	0.55	0.62	0.65
Paraguay	0.62	0.65	0.69	0.74	0.74	0.67	0.68
Peru	0.64	0.64	0.67	0.70	0.74	0.70	0.68
Romania	0.89	0.95	0.84	0.80	0.85	1.00	0.89
Russia	0.82	0.77	0.68	0.67	0.65	0.79	0.73
Serbia	0.87	0.91	0.80	0.80	0.80	0.81	0.83
South Africa	0.80	0.84	0.88	0.88	0.90	0.95	0.88
Thailand	0.76	0.87	0.84	0.83	0.93	0.81	0.84
Turkey	0.96	1.00	0.89	0.84	0.88	0.88	0.91
Turkmenistan	0.55	0.62	0.58	0.60	0.65	0.64	0.61
Venezuela	0.69	0.74	0.66	0.65	0.64	0.82	0.70
C) Productivity change
  Malmquist Index analysis results are presented in Table 5. The mean TFP change for the five years was 1.01, indicating a general increase in TFP over the study period. Although TFP declined from 2012 to 2014, the number of counties with raising efficiency (EC > 1) increased over the study period. TFP improved in 2011, 2013, and 2014 in Iran.

**Table 5:** Productivity changes of selected countries between 2010 and 2015

Country	2010–2011	2011–2012	2012–2013	2013–2014	2014–2015
Albania	1.06	0.99	1.02	1.02	0.95
Algeria	1.07	1.19	1.02	0.92	1.04
Argentina	1.05	1.17	1.04	1.03	0.64
Azerbaijan	0.99	1.03	1.00	1.02	0.93
Belarus	0.95	1.06	0.89	1.04	0.94
Bosnia and Herzegovina	0.92	1.00	1.04	0.95	0.94
Botswana	0.93	1.20	0.97	0.97	1.18
Brazil	1.01	1.02	1.07	1.01	0.92
Bulgaria	0.97	1.03	0.96	0.86	1.10
China	1.05	1.05	1.04	1.05	0.77
Colombia	1.09	1.05	0.96	0.91	0.92
Costa Rica	0.94	0.97	0.98	0.98	0.96
Cuba	0.93	1.17	0.86	0.80	1.41
Dominican Republic	1.07	1.03	1.01	1.09	0.86
Ecuador	1.03	1.06	1.14	1.12	0.74
Gabon	0.96	1.05	1.15	0.86	1.02
Iran	1.02	0.96	1.01	1.30	0.87
Iraq	1.00	1.08	1.11	0.90	0.88
Jamaica	0.99	1.05	1.02	0.95	1.16
Kazakhstan	0.98	1.09	0.97	1.12	0.97
Lebanon	1.00	1.10	0.97	0.99	1.01
Macedonia	1.01	1.03	1.02	1.00	0.95
Malaysia	0.99	1.06	1.03	1.08	1.02
Mexico	1.05	1.05	1.03	1.04	1.02
Namibia	1.02	0.99	1.00	1.06	1.04
Panama	1.00	0.93	0.79	0.94	1.17
Paraguay	1.05	1.07	1.08	0.99	0.91
Peru	1.00	1.05	1.05	1.05	0.94
Romania	1.00	0.96	0.94	0.99	1.24
Russia	0.94	0.89	0.98	0.97	1.24
Serbia	0.97	0.96	0.99	0.96	1.06
South Africa	1.05	1.05	1.01	1.02	1.06
Thailand	1.08	1.05	0.99	1.04	0.90
Turkey	0.96	0.98	0.94	0.97	1.04
Turkmenistan	1.03	1.01	1.04	1.00	1.03
Venezuela	1.07	0.90	0.98	0.99	1.29
D) Best performing countries (2010−2015)
  Our EDEA model identified four of ‘best performing’ countries with better performance related to their level of spending compared to other countries, i.e., Bosnia and Herzegovina, Namibia, Algeria, and Belarus (Fig. 2). Namibia had the highest level of spending (674 Int’l Dollar per capita), while the remaining three best-performing countries spent less than Namibia, ranging from 182 to 327 Int’l Dollar per capita). Regarding the level of spending for health, the reference country for Iran was Kazakhstan (Fig. 3).

**Fig. 2:**
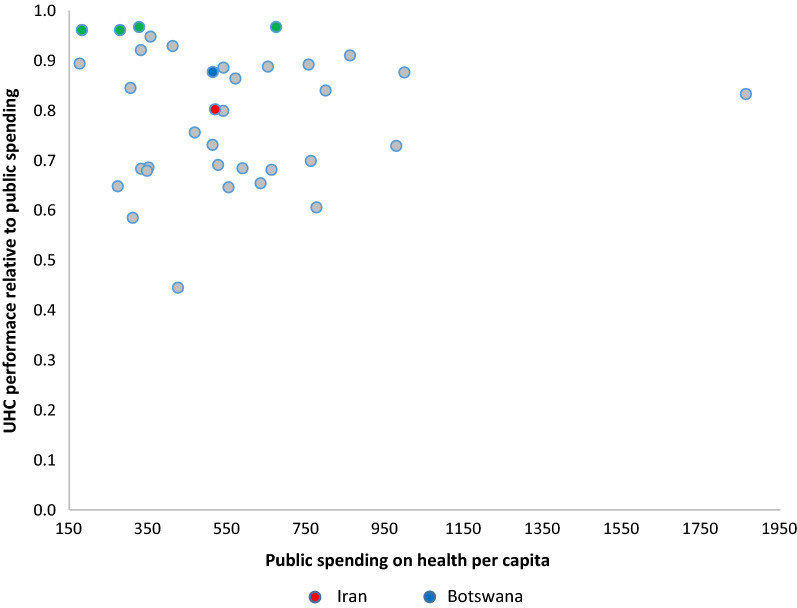
UHC performance relative to public spending in 36 selected countries

**Fig. 3:**
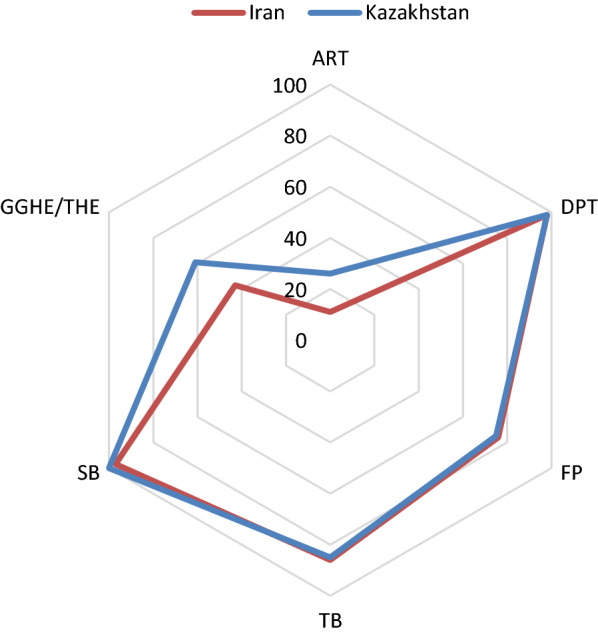
Comparison of UHC progress in Kazakhestan and Iran

## Discussion

This study aimed to measure the TE changes and productivity variations of Iran’s health system in making progress towards UHC over the 2010−2015 period. We employed a modified DEA method to measure TE changes and productivity variations. The DEA method is widely used in health system efficiency assessment; however, the efficiency assessment obtained by this method depends on the setting of outputs and inputs [42]. We chose our variables from UHC tracer indicators, as mentioned above. We found that the current indicators may not be well-suitable for monitoring the progress towards UHC, given that countries are at different stages of progress. Thus, additional UHC tracer indicators, representing different program areas of a health system and obtained from routine data that have shorter intervals in data gathering, are needed.

Our findings demonstrate slightly positive changes in efficiency scores in 2014 and 2015. We reviewed the changes that occurred in relevant input and output indicators as well as the recent HTP. Understandably, the three indicators of DPT3, FP, and SAB could not show change during the study period. This was because the data for these indicators were based on the surveys, which we do not expect their repetition in a few years’ intervals. The TB treatment success rate and at least a part of the ART coverage were based on routine data that we can expect changes, but it was not the case for the interval under study. Only two indicators of GGHE per capita and GGHE/THE changed over this period. Therefore, it seems that changes made in these two indicators, especially those made in the output indicator, have improved the efficiency score in 2014 and 2015, which are mostly attributable to the HTP implementation.

As the key health sector reform towards UHC in Iran [18, 43, 44], HTP aimed to increase the sustainability of health financing, expand health insurance coverage, increase financial protection against catastrophic health costs, and improve access to quality healthcare services. Several interventions have been implementing to provide additional financial resources and increase the existing ones, control the price of drugs and medical equipment, expand health insurance coverage among all society, reduce the share of inpatient and outpatient payments, prevent informal payments, increase the number of health facilities and workforce, and develop or revise the primary health care programs. It has been reported that, to some extent, HTP could resolve urgent challenges such as the high rise of OOP. However, further interventions are still required so that the Iranian health system can obtain better value for money that is to be spending on it. These include reforms to improve governance structures, financing arrangements, and service provision [19, 20, 45].

With the efficiency score of lower than average, our findings revealed Iran’s low rank among the studied countries. The efficiency model, as well as descriptive statistics of output indicators, suggest an equal to or higher than the average coverage of DPT3, TB, and SAB in Iran, compared to the studied countries over the study years. It demonstrates that a low score of Iran’s health system efficiency might be due to other factors than these indicators. Nevertheless, the status of the other three output indicators is different. FP coverage has slightly increased over the study years, which is significantly lower than the average of the studied countries and dramatically lower than the maximum value. A small increase is also observed in the ART coverage indicator in Iran, which is lower than the average of the studied countries. Although Iran’s public sector share of total health expenditure was significantly lower than the average of the studied countries from 2010 to 2013, it slightly increased between 2010 and 2013. Following the implementation of HTP, it increased significantly in 2014 and decreased again in 2015. Therefore, the low score of Iran’s efficiency can be attributed to these last three output indicators.

In addition, part of this low efficiency can be attributed to the changes that occurred in the input indicator. The public health expenditure per capita has declined to lower than the average of the studied countries over 2010 to 2013 [20]. This indicator increased significantly in 2014, rising even above the average of the studied countries, and then declined in 2015. Nonetheless, it is still higher than the indicators calculated in 2013 and before. Since output indicators have not changed significantly, the rising trend of this indicator makes it more expensive for Iran to meet the UHC goals. In other words, Iran’s health system has failed to efficiently use the new required resources to achieve UHC.

Many international and national policies have specifically emphasized the necessity of promoting the efficiency of health systems [24, 25, 46, 47]. Low efficiency and the waste of health resources are among the main challenges that the health systems in Iran [48] and other developing countries [49, 50] have been facing. In this study, we selected the efficiency indicators among relevant national and international indicators [51]. We recommend, while maintaining the increasing trend of UHC indicators, health policymakers in Iran need to follow three below interventions to improve the status of Iran’s health efficiency:

1. Interventions to expand the ART coverage: These interventions are suggested to be determined and prioritized using evidence-based practice models in order to identify and effectively cover high-risk groups through public health resources and improve effective HIV coverage.
2. Targeted interventions to improve universal financial coverage against ever-spiraling health costs at the health system and public levels. At the health system level, a significant part of terrible health costs can be attributed to the wide range of services covered by public resources, as well as the use of inefficient service delivery models. In Iran, a broad range of low cost-effective services have been covered through available public resources, without following any priority setting. Considering current constraints of financial health resources in Iran, covering some of these less-prioritized services through basic health insurance seems to be economically inefficient [52, 53]. Worse still, fee-for-service (FFS) based payment method has increased the risk of informal payments and induced demands, both of which have contributed to the increased share of direct out-of-pocket (OOP) payments, and catastrophic health costs, ultimately. Let alone, after 15 years into the implementation of the family physician program and the referral system, the national health system is still behind to establish appropriate rationing of services, which adds to the burden of undesirable health costs [18, 22, 54].
  At the public level, further evidence is needed to determine the health problems that impose high health costs. It can, in turn, help design and make targeted interventions to improve coverage against expensive healthcare services. Given the significant role of the private sector in Iran’s health system, particularly for hospital care, effective policies are essential for better engagement with the private sector, galvanized by empowering the community and raising public awareness about the appropriate and rational use of health services.
3. Sustainable interventions to allocate enough share of the general government budget to the health sector: The health sector’s share of government budget increased significantly following the implementation of HTP in 2014 [18, 48, 54, 55]. Given the current unilateral economic sanctions against Iran and the consecutive financial limits, serious concerns exist about the amount of financial resources allocated to the health sector as well as the timely allocation of such resources, if any [21]. Economic resiliency, for instance, through some interventions to reduce Iran’s dependency on oil revenue, as proposed in the proposed budget of 2020, could enhance financial stability and strengthen the health system sector. In particular, increasing sin taxes and tolls imposed on harmful products (e.g., tobacco products and soft drinks), plus the allocation of a larger share of value-added tax for the health sector, are helpful. Another example is the increasing share of health insurance resources. Analysis conducted following the economic shocks caused by sanctions and a sudden increase in the exchange rate in 2013 suggests that the most unstable financial resources in the health sector include direct OOP and government funds, respectively [56]. Hence, these two types of resources should be transferred into insurance funds. Finally, fundamental reforms are essential to eliminate or reduce conflict of interest in health-policy making, tailor the structure of health system, and improve payment system and benefits package design, aiming to tackle the main sources of waste of healthcare resources in Iran structures [20, 45, 57].

## Conclusion

There is a growing demand for efficiency improvements in the health systems to achieve UHC. While there is no defined set of indicators or precise method to measure health system efficiency, using some techniques such as EDEA might help to paint the picture of health system efficiency, which will be in turn a starting point to identify the causes of any inefficiencies and design tailored interventions for improving efficiency in any specific context. However, relying on the existing set of tracer indicators of UHC at the global level is not satisfactory for a country such as Iran to monitor the trend of the health system’s efficiency in achieving UHC. It needs indicators representing different program areas of a health system and indicators from routine data that have shorter intervals in data gathering. We found that Iran gained in the health system efficiency toward UHC from 2010 to 2015, which mainly achieved through better resource mobilization (CCHE per capita and GGHE/THE). More improvement in efficiency needs further reforms in the health system financing and also focusing on undermined program areas such as ART.

## Supplementary information

**Additional file 1.** Appendix—Input and output variables of selected countries for a period of six years (2010-2015).

## Data Availability

Not applicable.

## Abbreviations

AE: Allocative Efficiency
ART: Antiretroviral Therapy
DMUs: Decision-Making Units
DPT3: Diphtheria, Tetanus and Pertussis
EDEA: Extended Data Envelopment Analysis
FP: Family Planning
GGHE: General Government Health Expenditure
GHO: Global Health Observatory
HTP: Health Transformation Plan
LMICs: Middle Income Countries
MPI: Malmquist Productivity Index
NHA: National Health Accounts
OOP: Out Of Pocket
PHC: Primary Health Care
SAB: Skilled Attendants at Birth
TB: Tuberculosis
TE: Technical Efficiency
TFP: Total Factor Productivity
THE: Total Health Expenditure
UHC: Universal Health Coverage
UMICs: Upper Middle-Income Countries
WB: World Bank
WHO: World Health Organization
