# Supplementary material for: Assessing the efficiency of Iran health system in making progress towards universal health coverage: a comparative panel data analysis
Source: Cost Eff Resour Alloc. 2020 Jun 29;18:20. doi: 10.1186/s12962-020-00215-x (PMC7324989; doi:10.1186/s12962-020-00215-x)
Supplement: Supplementary file 1 — Additional file 1. Appendix—Input and output variables of selected countries for a period of six years (2010-2015). [file 12962_2020_215_MOESM1_ESM.docx]

**Additional file**

| Countries  2010 | Antiretroviral Therapy coverage | Diphtheria, Tetanus and Pertussis coverage | Family Planning coverage | Tuberculosis success rate | Skilled Attendants at Birth coverage | General Government Health Expenditure as % of Total Health Expenditure | General Government Health Expenditure (GGHE) per capita (International dollar) |
| --- | --- | --- | --- | --- | --- | --- | --- |
| Albania | 12 | 99 | 19 | 89 | 99 | 46 | 231 |
| Algeria | 24 | 95 | 75 | 89 | 95 | 70 | 452 |
| Argentina | 55 | 94 | 81 | 45 | 95 | 64 | 817 |
| Azerbaijan | 10 | 81 | 27 | 73 | 99 | 22 | 182 |
| Belarus | 29 | 98 | 71 | 78 | 100 | 78 | 664 |
| Bosnia and Herzegovina | 21 | 89 | 23 | 98 | 100 | 71 | 607 |
| Botswana | 50 | 95 | 76 | 78 | 99 | 63 | 490 |
| Brazil | 38 | 99 | 88 | 72 | 99 | 46 | 534 |
| Bulgaria | 12 | 94 | 53 | 87 | 100 | 56 | 606 |
| China | 21 | 99 | 95 | 95 | 100 | 54 | 245 |
| Colombia | 20 | 88 | 82 | 74 | 95 | 74 | 531 |
| Costa Rica | 45 | 88 | 91 | 88 | 95 | 73 | 880 |
| Cuba | 54 | 96 | 88 | 90 | 100 | 95 | 1763 |
| Dominican Republic | 22 | 88 | 84 | 80 | 97 | 56 | 249 |
| Ecuador | 28 | 91 | 81 | 76 | 89 | 42 | 225 |
| Gabon | 22 | 67 | 32 | 60 | 89 | 72 | 386 |
| Iran | 3 | 99 | 74 | 83 | 96 | 34 | 415 |
| Iraq | 21 | 74 | 59 | 90 | 85 | 74 | 289 |
| Jamaica | 20 | 94 | 83 | 36 | 98 | 56 | 241 |
| Kazakhstan | 8 | 99 | 74 | 76 | 99 | 57 | 486 |
| Lebanon | 27 | 81 | 59 | 81 | 99 | 38 | 432 |
| Macedonia | 17 | 95 | 23 | 85 | 100 | 63 | 501 |
| Malaysia | 12 | 96 | 52 | 76 | 99 | 57 | 459 |
| Mexico | 36 | 95 | 82 | 82 | 94 | 49 | 473 |
| Namibia | 43 | 83 | 73 | 85 | 84 | 100 | 321 |
| Panama | 32 | 94 | 70 | 75 | 93 | 70 | 839 |
| Paraguay | 19 | 89 | 80 | 70 | 96 | 37 | 240 |
| Peru | 29 | 93 | 62 | 68 | 84 | 56 | 270 |
| Romania | 55 | 94 | 65 | 84 | 100 | 80 | 775 |
| Russia | 21 | 97 | 70 | 66 | 100 | 54 | 756 |
| Serbia | 57 | 91 | 34 | 88 | 100 | 62 | 738 |
| South Africa | 20 | 72 | 83 | 53 | 92 | 47 | 465 |
| Thailand | 41 | 99 | 92 | 83 | 99 | 75 | 355 |
| Turkey | 21 | 97 | 58 | 89 | 93 | 79 | 711 |
| Turkmenistan | 21 | 96 | 73 | 87 | 99 | 62 | 120 |
| Venezuela | 41 | 78 | 81 | 82 | 98 | 41 | 339 |

| Countries  2011 | Antiretroviral Therapy coverage | Diphtheria, Tetanus and Pertussis coverage | Family Planning coverage | Tuberculosis success rate | Skilled Attendants at Birth coverage | General Government Health Expenditure as % of Total Health Expenditure | General Government Health Expenditure (GGHE) per capita (International dollar) |
| --- | --- | --- | --- | --- | --- | --- | --- |
| Albania | 14 | 99 | 21 | 92 | 99 | 50 | 276 |
| Algeria | 28 | 95 | 75 | 92 | 96 | 71 | 490 |
| Argentina | 56 | 91 | 81 | 49 | 97 | 64 | 780 |
| Azerbaijan | 15 | 87 | 28 | 78 | 97 | 22 | 169 |
| Belarus | 30 | 98 | 72 | 71 | 100 | 71 | 576 |
| Bosnia and Herzegovina | 24 | 88 | 23 | 72 | 100 | 71 | 652 |
| Botswana | 54 | 95 | 76 | 76 | 100 | 62 | 430 |
| Brazil | 40 | 99 | 88 | 73 | 99 | 45 | 542 |
| Bulgaria | 14 | 95 | 55 | 86 | 100 | 55 | 585 |
| China | 24 | 99 | 95 | 95 | 100 | 56 | 288 |
| Colombia | 28 | 85 | 83 | 73 | 99 | 76 | 577 |
| Costa Rica | 46 | 85 | 90 | 88 | 98 | 73 | 935 |
| Cuba | 58 | 97 | 88 | 88 | 100 | 95 | 1906 |
| Dominican Republic | 27 | 84 | 84 | 83 | 98 | 62 | 296 |
| Ecuador | 34 | 88 | 81 | 73 | 91 | 44 | 258 |
| Gabon | 26 | 75 | 33 | 48 | 90 | 67 | 353 |
| Iran | 4 | 99 | 75 | 84 | 96 | 35 | 432 |
| Iraq | 24 | 79 | 59 | 90 | 91 | 75 | 279 |
| Jamaica | 23 | 92 | 83 | 44 | 99 | 54 | 233 |
| Kazakhstan | 11 | 99 | 74 | 76 | 100 | 56 | 472 |
| Lebanon | 30 | 81 | 59 | 78 | 99 | 39 | 430 |
| Macedonia | 20 | 96 | 23 | 89 | 91 | 65 | 497 |
| Malaysia | 13 | 96 | 52 | 76 | 99 | 55 | 454 |
| Mexico | 38 | 97 | 82 | 82 | 95 | 51 | 505 |
| Namibia | 51 | 82 | 74 | 86 | 85 | 100 | 337 |
| Panama | 36 | 87 | 71 | 81 | 94 | 68 | 840 |
| Paraguay | 19 | 89 | 80 | 71 | 96 | 40 | 283 |
| Peru | 31 | 91 | 63 | 74 | 85 | 53 | 272 |
| Romania | 55 | 89 | 67 | 86 | 99 | 79 | 770 |
| Russia | 24 | 97 | 71 | 65 | 99 | 54 | 807 |
| Serbia | 58 | 94 | 35 | 86 | 99 | 62 | 759 |
| South Africa | 27 | 69 | 84 | 77 | 93 | 48 | 501 |
| Thailand | 45 | 99 | 92 | 82 | 99 | 78 | 408 |
| Turkey | 24 | 97 | 59 | 90 | 93 | 80 | 749 |
| Turkmenistan | 24 | 97 | 73 | 87 | 99 | 64 | 144 |
| Venezuela | 41 | 78 | 81 | 80 | 98 | 45 | 397 |

| Countries  2012 | Antiretroviral Therapy coverage | Diphtheria, Tetanus and Pertussis coverage | Family Planning coverage | Tuberculosis success rate | Skilled Attendants at Birth coverage | General Government Health Expenditure as % of Total Health Expenditure | General Government Health Expenditure (GGHE) per capita (International dollar) |
| --- | --- | --- | --- | --- | --- | --- | --- |
| Albania | 17 | 99 | 22 | 92 | 99 | 49 | 266 |
| Algeria | 33 | 95 | 75 | 90 | 97 | 74 | 604 |
| Argentina | 58 | 91 | 83 | 56 | 98 | 59 | 668 |
| Azerbaijan | 19 | 89 | 29 | 83 | 100 | 23 | 197 |
| Belarus | 34 | 98 | 72 | 85 | 100 | 77 | 663 |
| Bosnia and Herzegovina | 28 | 92 | 24 | 84 | 100 | 71 | 675 |
| Botswana | 61 | 95 | 77 | 76 | 100 | 63 | 554 |
| Brazil | 43 | 95 | 88 | 72 | 99 | 44 | 557 |
| Bulgaria | 16 | 95 | 56 | 87 | 100 | 56 | 637 |
| China | 28 | 99 | 95 | 95 | 100 | 56 | 329 |
| Colombia | 33 | 91 | 83 | 72 | 99 | 76 | 636 |
| Costa Rica | 46 | 91 | 90 | 86 | 99 | 73 | 968 |
| Cuba | 59 | 99 | 88 | 85 | 100 | 94 | 1605 |
| Dominican Republic | 31 | 85 | 84 | 82 | 98 | 63 | 317 |
| Ecuador | 34 | 87 | 81 | 75 | 93 | 46 | 310 |
| Gabon | 31 | 82 | 34 | 54 | 89 | 71 | 393 |
| Iran | 6 | 99 | 75 | 87 | 96 | 35 | 401 |
| Iraq | 28 | 69 | 60 | 91 | 86 | 62 | 406 |
| Jamaica | 26 | 96 | 83 | 65 | 98 | 58 | 278 |
| Kazakhstan | 14 | 99 | 74 | 86 | 100 | 56 | 526 |
| Lebanon | 33 | 81 | 59 | 71 | 99 | 46 | 503 |
| Macedonia | 25 | 95 | 24 | 86 | 100 | 65 | 525 |
| Malaysia | 14 | 97 | 52 | 78 | 99 | 55 | 498 |
| Mexico | 43 | 99 | 83 | 80 | 100 | 51 | 535 |
| Namibia | 55 | 84 | 75 | 85 | 86 | 100 | 323 |
| Panama | 37 | 85 | 72 | 80 | 94 | 69 | 906 |
| Paraguay | 22 | 87 | 80 | 70 | 96 | 44 | 342 |
| Peru | 40 | 95 | 63 | 67 | 87 | 55 | 314 |
| Romania | 56 | 91 | 68 | 85 | 100 | 80 | 807 |
| Russia | 28 | 97 | 72 | 69 | 100 | 55 | 912 |
| Serbia | 58 | 91 | 35 | 84 | 99 | 61 | 775 |
| South Africa | 34 | 65 | 84 | 77 | 93 | 49 | 534 |
| Thailand | 49 | 99 | 91 | 81 | 100 | 78 | 447 |
| Turkey | 28 | 97 | 59 | 88 | 94 | 79 | 752 |
| Turkmenistan | 28 | 97 | 73 | 84 | 99 | 65 | 162 |
| Venezuela | 42 | 81 | 81 | 82 | 96 | 35 | 302 |

| Countries  2013 | Antiretroviral Therapy coverage | Diphtheria, Tetanus and Pertussis coverage | Family Planning coverage | Tuberculosis success rate | Skilled Attendants at Birth coverage | General Government Health Expenditure as % of Total Health Expenditure | General Government Health Expenditure (GGHE) per capita (International dollar) |
| --- | --- | --- | --- | --- | --- | --- | --- |
| Albania | 20 | 99 | 23 | 88 | 99 | 50 | 283 |
| Algeria | 43 | 95 | 76 | 91 | 97 | 73 | 625 |
| Argentina | 60 | 94 | 85 | 51 | 97 | 55 | 644 |
| Azerbaijan | 23 | 93 | 30 | 82 | 100 | 21 | 197 |
| Belarus | 36 | 98 | 73 | 87 | 100 | 66 | 708 |
| Bosnia and Herzegovina | 32 | 89 | 25 | 82 | 100 | 70 | 644 |
| Botswana | 65 | 95 | 77 | 73 | 100 | 60 | 537 |
| Brazil | 47 | 97 | 88 | 72 | 99 | 45 | 602 |
| Bulgaria | 19 | 95 | 57 | 85 | 100 | 52 | 661 |
| China | 32 | 99 | 95 | 95 | 100 | 56 | 365 |
| Colombia | 39 | 91 | 83 | 71 | 99 | 76 | 663 |
| Costa Rica | 45 | 95 | 90 | 88 | 99 | 73 | 993 |
| Cuba | 60 | 99 | 88 | 84 | 100 | 95 | 1884 |
| Dominican Republic | 34 | 83 | 84 | 83 | 99 | 64 | 323 |
| Ecuador | 36 | 87 | 81 | 75 | 95 | 53 | 422 |
| Gabon | 41 | 79 | 35 | 55 | 90 | 74 | 494 |
| Iran | 7 | 98 | 75 | 87 | 96 | 39 | 408 |
| Iraq | 32 | 68 | 61 | 88 | 86 | 63 | 487 |
| Jamaica | 28 | 93 | 83 | 77 | 98 | 57 | 294 |
| Kazakhstan | 19 | 98 | 75 | 89 | 100 | 51 | 506 |
| Lebanon | 36 | 81 | 60 | 71 | 99 | 46 | 476 |
| Macedonia | 28 | 98 | 25 | 91 | 100 | 69 | 524 |
| Malaysia | 16 | 97 | 52 | 76 | 99 | 55 | 519 |
| Mexico | 45 | 83 | 83 | 80 | 96 | 52 | 554 |
| Namibia | 58 | 89 | 75 | 86 | 88 | 100 | 322 |
| Panama | 43 | 80 | 73 | 80 | 91 | 72 | 1144 |
| Paraguay | 25 | 86 | 80 | 68 | 91 | 46 | 407 |
| Peru | 41 | 88 | 63 | 79 | 89 | 58 | 356 |
| Romania | 59 | 92 | 69 | 85 | 99 | 81 | 865 |
| Russia | 32 | 97 | 72 | 68 | 100 | 52 | 929 |
| Serbia | 62 | 95 | 35 | 78 | 100 | 59 | 779 |
| South Africa | 40 | 73 | 84 | 78 | 93 | 48 | 538 |
| Thailand | 53 | 99 | 91 | 81 | 99 | 77 | 443 |
| Turkey | 32 | 98 | 60 | 86 | 97 | 78 | 790 |
| Turkmenistan | 32 | 98 | 73 | 72 | 99 | 67 | 200 |
| Venezuela | 40 | 82 | 81 | 81 | 98 | 31 | 282 |

| Countries  2014 | Antiretroviral Therapy coverage | Diphtheria, Tetanus and Pertussis coverage | Family Planning coverage | Tuberculosis success rate | Skilled Attendants at Birth coverage | General Government Health Expenditure as % of Total Health Expenditure | General Government Health Expenditure (GGHE) per capita (International dollar) |
| --- | --- | --- | --- | --- | --- | --- | --- |
| Albania | 23 | 98 | 24 | 88 | 99 | 50 | 307 |
| Algeria | 53 | 95 | 76 | 88 | 96 | 73 | 678 |
| Argentina | 61 | 94 | 85 | 52 | 100 | 55 | 630 |
| Azerbaijan | 29 | 94 | 31 | 83 | 100 | 20 | 214 |
| Belarus | 36 | 97 | 73 | 88 | 100 | 66 | 678 |
| Bosnia and Herzegovina | 36 | 86 | 26 | 77 | 100 | 71 | 681 |
| Botswana | 70 | 95 | 78 | 77 | 99 | 59 | 514 |
| Brazil | 51 | 93 | 88 | 71 | 99 | 46 | 607 |
| Bulgaria | 21 | 88 | 58 | 86 | 100 | 55 | 763 |
| China | 36 | 99 | 95 | 94 | 100 | 56 | 408 |
| Colombia | 45 | 90 | 83 | 76 | 99 | 75 | 723 |
| Costa Rica | 45 | 91 | 90 | 89 | 99 | 73 | 1010 |
| Cuba | 65 | 99 | 88 | 82 | 99 | 96 | 2366 |
| Dominican Republic | 39 | 91 | 84 | 83 | 98 | 67 | 388 |
| Ecuador | 42 | 83 | 81 | 77 | 96 | 49 | 512 |
| Gabon | 45 | 70 | 36 | 58 | 91 | 68 | 410 |
| Iran | 9 | 99 | 76 | 87 | 96 | 50 | 646 |
| Iraq | 36 | 64 | 61 | 92 | 87 | 60 | 402 |
| Jamaica | 31 | 92 | 83 | 18 | 98 | 52 | 249 |
| Kazakhstan | 23 | 95 | 75 | 90 | 100 | 54 | 581 |
| Lebanon | 40 | 81 | 60 | 76 | 99 | 48 | 470 |
| Macedonia | 33 | 95 | 27 | 87 | 100 | 63 | 539 |
| Malaysia | 21 | 97 | 52 | 78 | 99 | 55 | 574 |
| Mexico | 47 | 87 | 83 | 80 | 96 | 52 | 581 |
| Namibia | 59 | 88 | 76 | 87 | 87 | 100 | 375 |
| Panama | 45 | 80 | 73 | 79 | 94 | 73 | 1228 |
| Paraguay | 28 | 87 | 80 | 71 | 92 | 46 | 400 |
| Peru | 47 | 88 | 63 | 87 | 90 | 61 | 398 |
| Romania | 63 | 94 | 70 | 85 | 99 | 80 | 868 |
| Russia | 36 | 97 | 73 | 69 | 100 | 52 | 958 |
| Serbia | 62 | 93 | 35 | 81 | 98 | 62 | 812 |
| South Africa | 45 | 77 | 84 | 78 | 94 | 48 | 554 |
| Thailand | 58 | 99 | 91 | 80 | 99 | 78 | 467 |
| Turkey | 36 | 96 | 60 | 87 | 97 | 77 | 803 |
| Turkmenistan | 36 | 98 | 73 | 84 | 99 | 65 | 209 |
| Venezuela | 39 | 78 | 81 | 80 | 100 | 29 | 271 |

| Countries  2015 | Antiretroviral Therapy coverage | Diphtheria, Tetanus and Pertussis coverage | Family Planning coverage | Tuberculosis success rate | Skilled Attendants at Birth coverage | General Government Health Expenditure as % of Total Health Expenditure | General Government Health Expenditure (GGHE) per capita (International dollar) |
| --- | --- | --- | --- | --- | --- | --- | --- |
| Albania | 26 | 99 | 25 | 90 | 99 | 42 | 271 |
| Algeria | 65 | 95 | 76 | 91 | 96 | 75 | 573 |
| Argentina | 63 | 94 | 85 | 53 | 99 | 60 | 994 |
| Azerbaijan | 34 | 96 | 32 | 81 | 99 | 20 | 133 |
| Belarus | 42 | 99 | 74 | 84 | 100 | 75 | 753 |
| Bosnia and Herzegovina | 41 | 82 | 27 | 87 | 100 | 63 | 547 |
| Botswana | 77 | 95 | 78 | 77 | 99 | 67 | 636 |
| Brazil | 57 | 96 | 88 | 74 | 99 | 46 | 688 |
| Bulgaria | 24 | 91 | 59 | 87 | 100 | 64 | 720 |
| China | 41 | 99 | 95 | 95 | 99 | 48 | 193 |
| Colombia | 53 | 91 | 83 | 75 | 96 | 76 | 787 |
| Costa Rica | 45 | 92 | 90 | 88 | 99 | 76 | 1079 |
| Cuba | 69 | 99 | 88 | 87 | 100 | 94 | 1655 |
| Dominican Republic | 46 | 85 | 84 | 83 | 98 | 45 | 289 |
| Ecuador | 50 | 78 | 82 | 77 | 95 | 39 | 260 |
| Gabon | 56 | 80 | 37 | 57 | 91 | 51 | 434 |
| Iran | 11 | 98 | 76 | 86 | 97 | 43 | 504 |
| Iraq | 41 | 58 | 62 | 91 | 87 | 69 | 239 |
| Jamaica | 32 | 91 | 83 | 49 | 98 | 58 | 379 |
| Kazakhstan | 26 | 98 | 75 | 85 | 100 | 61 | 545 |
| Lebanon | 44 | 81 | 61 | 77 | 99 | 41 | 766 |
| Macedonia | 41 | 91 | 28 | 88 | 99 | 65 | 733 |
| Malaysia | 26 | 99 | 53 | 78 | 99 | 59 | 578 |
| Mexico | 55 | 87 | 83 | 82 | 96 | 49 | 591 |
| Namibia | 63 | 92 | 77 | 87 | 87 | 93 | 455 |
| Panama | 48 | 73 | 74 | 80 | 95 | 71 | 1034 |
| Paraguay | 30 | 93 | 81 | 72 | 92 | 42 | 318 |
| Peru | 53 | 90 | 64 | 77 | 84 | 58 | 351 |
| Romania | 67 | 89 | 71 | 86 | 100 | 81 | 710 |
| Russia | 41 | 97 | 73 | 69 | 100 | 63 | 803 |
| Serbia | 63 | 95 | 36 | 85 | 99 | 67 | 793 |
| South Africa | 49 | 75 | 84 | 75 | 94 | 47 | 649 |
| Thailand | 61 | 99 | 91 | 82 | 99 | 69 | 431 |
| Turkey | 41 | 97 | 60 | 89 | 94 | 75 | 763 |
| Turkmenistan | 41 | 99 | 74 | 84 | 99 | 68 | 221 |
| Venezuela | 55 | 87 | 82 | 82 | 98 | 42 | 488 |
